# cBid, Bax and Bcl-xL exhibit opposite membrane remodeling activities

**DOI:** 10.1038/cddis.2016.34

**Published:** 2016-02-25

**Authors:** S Bleicken, G Hofhaus, B Ugarte-Uribe, R Schröder, A J García-Sáez

**Affiliations:** 1 Membrane Biophysics, Max Planck Institute for Intelligent Systems, Heisenbergstrasse 3, Stuttgart 70569, Germany; 2 German Cancer Research Center, Im Neuenheimer Feld 267, Heidelberg 69120, Germany; 3 Membrane Biophysics, Interfaculty Institute of Biochemistry, Eberhard Karls University Tübingen, Hoppe-Seyler-Strasse 4, Tübingen 72076, Germany; 4 CellNetworks, Bioquant, Heidelberg University, Im Neuenheimer Feld 267, Heidelberg, 69120, Germany

## Abstract

The proteins of the Bcl-2 family have a crucial role in mitochondrial outer membrane permeabilization during apoptosis and in the regulation of mitochondrial dynamics. Current models consider that Bax forms toroidal pores at mitochondria that are responsible for the release of cytochrome c, whereas Bcl-xL inhibits pore formation. However, how Bcl-2 proteins regulate mitochondrial fission and fusion remains poorly understood. By using a systematic analysis at the single vesicle level, we found that cBid, Bax and Bcl-xL are able to remodel membranes in different ways. cBid and Bax induced a reduction in vesicle size likely related to membrane tethering, budding and fission, besides membrane permeabilization. Moreover, they are preferentially located at highly curved membranes. In contrast, Bcl-xL not only counterbalanced pore formation but also membrane budding and fission. Our findings support a mechanism of action by which cBid and Bax induce or stabilize highly curved membranes including non-lamellar structures. This molecular activity reduces the energy for membrane remodeling, which is a necessary step in toroidal pore formation, as well as membrane fission and fusion, and provides a common mechanism that links the two main functions of Bcl-2 proteins.

Apoptosis is a form of programmed cell death that removes unhealthy or unnecessary cells from multi-cellular organisms.^1, 2, 3^ The mitochondrial pathway of apoptosis results in the permeabilization of the mitochondrial outer membrane (MOM) and in the fragmentation of the tubular mitochondrial network. The members of the Bcl-2 family are crucial in both processes.^4, 5, 6, 7, 8, 9, 10^ They are classified into the following three sub-groups: the pro-apoptotic Bax-type proteins that mediate MOM permeabilization; the anti-apoptotic family members that promote cell survival by inhibiting their pro-apoptotic counterparts; and the BH3-only proteins that promote apoptosis either by inhibiting the anti-apoptotic Bcl-2 proteins or by activating Bax and Bak.^7, 10^

Here, we focused on cBid, Bax and Bcl-xL as representative members of all three subgroups. They shuttle between soluble and MOM-inserted conformations.^11, 12, 13, 14^ Apoptosis induction leads to their extended association with the mitochondrial membrane, where Bax and Bcl-xL adopt membrane-embedded conformations,^14, 15, 16, 17, 18, 19, 20, 21, 22, 23^ and associate into homo- or hetero-dimers.^15, 16, 17, 21, 24, 25, 26, 27^ Moreover, the Bcl-2 proteins are important regulators of mitochondrial morphology.^6, 28, 29, 30, 31^ Although active Bax promotes mitochondrial fission during apoptosis,^6, 28, 29, 30, 32^ inactive Bax is required for the normal rate of mitochondrial fusion.^29^ Interestingly, Bax co-localizes with proteins involved in mitochondrial fission and fusion (Drp1 and Mfn2).^28^ In addition, Bcl-xL impacts on mitochondrial fission and fusion,^31^ and stimulates the GTPase activity of Drp1.^33, 34^ However, the molecular mechanism underlying these functions remains obscure.

Membrane fission and fusion are lipid-mediated events that proceed through highly curved and energetically unfavorable, non-lamellar membrane structures.^35, 36, 37, 38^ The formation of toroidal pores, like those formed by Bax and Bak,^39, 40, 41, 42, 43, 44^ involves similar structures.^45, 46^ As the Bcl-2 proteins affect both toroidal pore formation and mitochondrial dynamics, it is a reasonable hypothesis that both processes are promoted by a common underlying activity. However, the relationship between these membrane structures and the Bcl-2 proteins remains enigmatic. To tackle this issue, reconstituted model systems have proven useful to learn about the molecular mechanisms involved.

Here, we used minimal systems to investigate how Bcl-2 proteins affect the organization of lipid membranes mimicking the MOM and specifically membrane remodeling. Our results show that cBid alone, cBid/Bax or heat-activated Bax are able to induce or stabilize curved structures, whereas permeabilization requires the presence of Bax. These alterations are specific, as they are abolished by Bcl-xL. Our findings suggest that the functional roles of Bcl-2 proteins in MOM permeabilization and mitochondrial shape are linked by their ability to stabilize unfavorable membrane structures.

## Results

We previously used model membranes to understand the interaction of Bcl-2 proteins with membranes.^14, 15, 16, 24, 43^ These systems recapitulate the main traits of the biological process, while decreasing its complexity so that aspects like pore formation or membrane fusion and fission can be studied in more detail than it is possible in cells. Here, we systematically investigated the effect of different Bcl-2 proteins on membrane structure. As the biophysical methods used have different requirements in terms of membrane amounts, we have kept the protein to lipid ratio constant to make the experiments comparable (Bax to lipid ratio ~1 : 500–1000; cBid to Bax ratio 1 : 2; Bax to Bcl-xL ratio 2 : 5). Unless otherwise stated, we used a lipid mixture mimicking the MOM that is well established in the field.^16, 21, 47^

Giant unilamellar vesicle (GUV) permeabilization was followed by the entry of soluble, fluorescently labeled proteins into the liposomes (introduced in Bleicken *et al.*^43^). After incubation in absence or presence of Bcl-2 proteins, the vesicle suspensions were imaged and analyzed with a home-built software^48^ to quantify GUV size, shape and permeabilization. To detect membrane remodeling, we analyzed the median radius of the GUVs and normalized it by the median radius of the control GUVs . The radii of the GUVs considered in the experiments ranged between 2 and 50 *μ*m. Smaller GUVs could not be detected due to the resolution of the method, whereas bigger GUVs were rare. The impact of different Bcl-2 proteins is expressed relative to the control.

**Figure 1:**
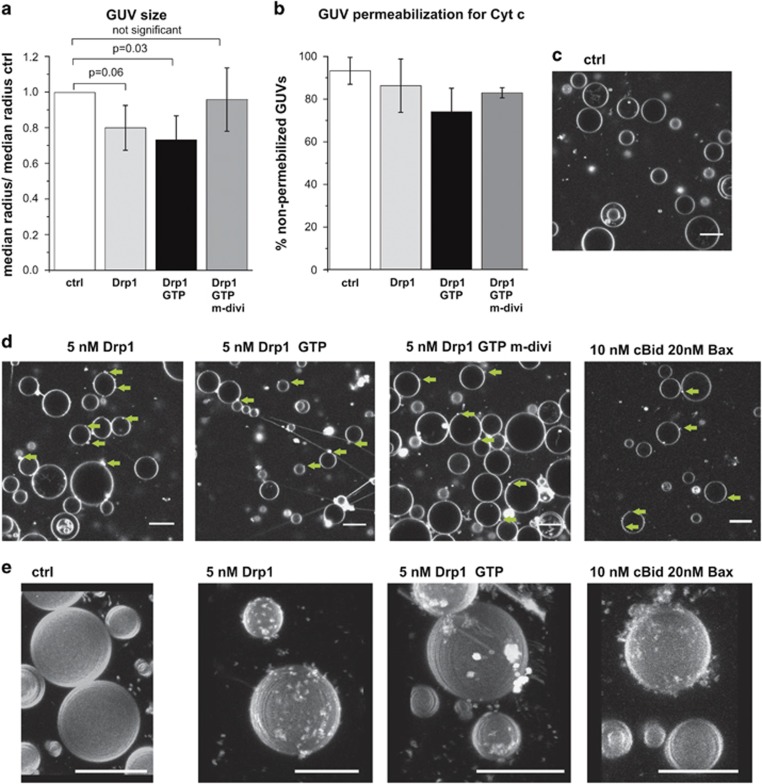
Changes in GUV size upon incubation with Drp1. (**a**) Comparison of the median GUV radius from GUV incubated 90 min with only Cyt c_488_ (control) or additionally with 5 nM Drp1, 1 mM GTP or 50 *μ*m mdivi-1 normalized to the median radius of the control sample. Notably, mdivi-1 partly precipitated, thus the effective mdivi-1 concentration is lower as 50 *μ*M. Error bars in (**a**) and (**b**) correspond to the S.D. (*N*=3). (**b**) Percentage of non-permeabilized GUVs for Cyt c_488_ from (**a**). (**c**–**e**) Confocal images (**c** and **d**) and 3D reconstitution of z-stacks from GUV incubated with the size marker proteins alone or with the components indicated in the figure. In all experiments the GUVs were composed of a lipid mixture mimicking the MOM and labeled with <0.05% DiI. Green arrows in (**d**) indicate structures indicative for vesicle buds. Scale bar: 20 *μ*m

**Figure 2:**
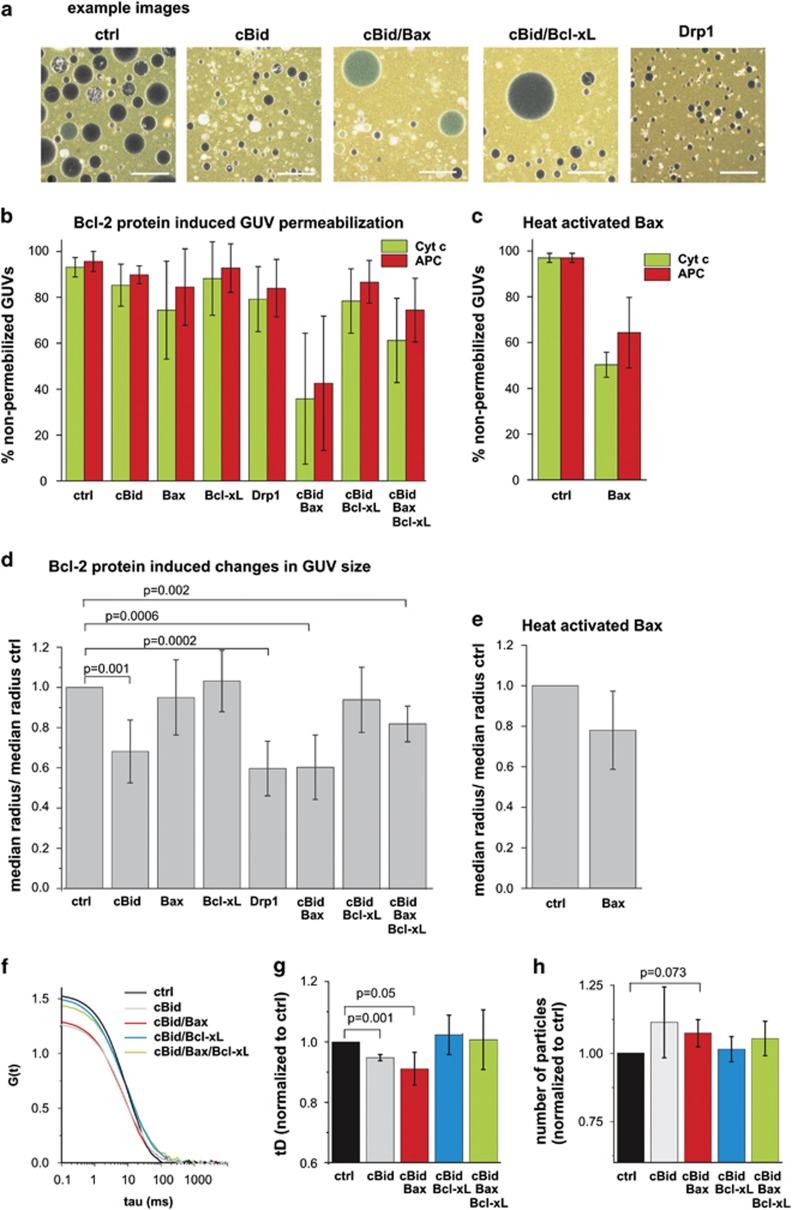
Changes in GUV size and membrane permeabilization induced by Bcl-2 proteins. (**a**) Representative images of GUVs (gray) in a solution of Cyt c_488_ (12 kDa, green) and allophycocianin (APC) (104 kDa, red) incubated in the absence (ctrl) or in the presence of 10 nM cBid, 20 nM Bax, 50 nM Bcl-xL, 10 nM Drp-1 or combinations of these proteins. Scale bar: 75 *μ*m. (**b** and **c**) Fraction of non-permeabilized GUVs in the control sample or in presence of the indicated proteins or protein mixture (**b**) or after heat treatment (**c**). Green and red bars correspond to Cyt c_488_ and APC, respectively. (**d** and **e**) Comparison of the median GUV radius from the experiments introduced in (**b** and **c**) normalized to the median radius of the control sample (4.97±1.07 *μ*m). (**d**) Results for individual proteins or protein mixtures. (**e**) Results from heat-treated samples. In each of four independent experiments, a minimum of 200 vesicles were analyzed per condition. Error bars represent the S.D. (**f**–**h**) Fluorescence correlation spectroscopy experiments on vesicle diffusion after 2 h incubation in buffer or with the indicated Bcl-2 proteins. Error bars present the S.D. (**f**) Auto-correlation curves from one exemplary experiment. The color code is indicated within the figure. (**g** and **h**) Comparison of the vesicle diffusion times (tD) (**g**) and particle numbers (**h**) from three independent experiments normalized to the tD or particle number of the control vesicles. Error bars correspond to the S.D. To achieve the tD a 3D fit was used

To validate the method, the influence of Drp1 on the GUV radius was tested. *In vivo*, Drp1 is involved in mitochondrial fission^49, 50^ and *in vitro* it induces tube formation and vesicle tethering.^40, 51^ Both changes extract membrane material and are expected to lead to a reduction in GUV size, as detected experimentally (Figures 1a and c–e for 5 nM Drp1; Figure 2d for 10 nM Drp1). Drp1 induced the formation of membrane tubes and buds on the GUV surface, which contrasted with the more homogeneous surface of control GUVs (Figures 1c and e). These effects were specific for Drp1, as GTP addition slightly enhanced GUV size reduction induced by Drp1, whereas addition of the Drp1 inhibitor mdivi-1^52, 53^ decreased the effects (Figures 1a, d and e). We did not detect vesicle bursting neither did Drp1 induce GUV permeabilization (Figure 1b), demonstrating that permeabilization and size reduction are not necessarily coupled.

### cBid and Bax cause membrane permeabilization and a reduction in GUV size, while both processes can be inhibited by Bcl-xL

**Figure 3:**
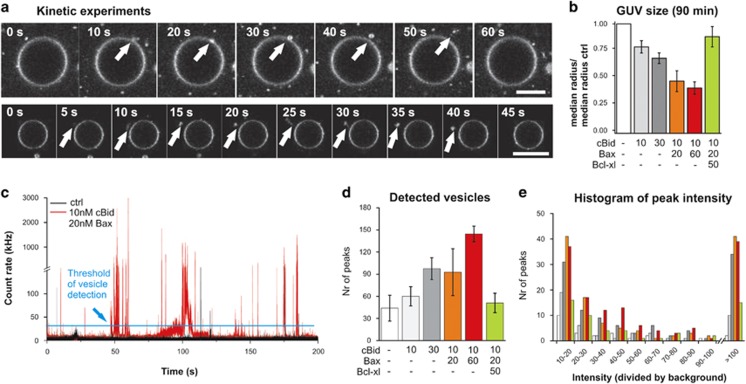
GUV budding upon incubation with Bcl-2 proteins. (**a**) Snapshots of vesicle budding from kinetic experiments. GUV mimicking the MOM composition labeled with DiI incubated with 10 nM cBid and 20 nM Bax. Scale bar: 20 *μ*m. (**b**) Changes in GUV size upon 90 min incubation with the indicated proteins at 22 °C. (**c**) Exemplary fluorescence intensity trace of a control sample and a sample incubated with 10 nM cBid and 20 nM Bax. The background count rate is <3 kHz. Fluorescent bursts >10 times brighter as the background count rate are considered as diffusing vesicles. (**d**) Number of peaks detected during 600 s (with count rates >29 kHz) after 90 min incubation with the indicated proteins at 22 °C. Error bar present the S.D. (*N*=3). (**e**) Histogram of the different peak intensities describes in (**d**). The color code is the same as in (**d**)

We then investigated the effect of individual Bcl-2 proteins on GUV size. Bax or Bcl-xL alone do not spontaneously bind to the membranes in the absence of cBid^21, 24, 26^ and thus they did not affect GUV size nor did they induce membrane permeabilization (Figures 2b and d). However, cBid mixed with Bax caused a significant reduction in GUV size and membrane permeabilization (Figures 2b, d and 3b).

Surprisingly, also cBid alone caused a concentration-dependent reduction of GUV size (Figures 2d and 3b), whereas no significant membrane permeabilization was detected (Figures 2a and b). The effect of cBid was specific, as Bcl-xL completely neutralized this effect despite enhancing cBid membrane binding^24, 26^ (Figures 2d and 3b). In presence of all three proteins, mild membrane permeabilization and size reduction was detected (Figures 2b, d and 3b), showing that Bcl-xL strongly counterbalanced the effects of cBid and Bax in line with its inhibitory role.

To separate the membrane effects of Bax and cBid, we tested an alternative method to activate Bax using mild heat (43 °C).^48, 54^ This treatment led to substantial membrane permeabilization in presence of Bax, but not in the control (Figure 2c), similar to our observations with cBid-induced Bax pores^43^ (Figure 2b). Moreover, heat-activated Bax also reduced the median GUV size (Figure 2e).

In addition, we used an alternative assay to detect vesicle size reduction based on large unilamellar vesicles (LUVs) and fluorescence correlation spectroscopy (FCS). The diffusion of LUVs through the focal volume of the confocal microscope is detected and analyzed by an auto-correlation analysis. The vesicle diffusion time, which directly relates to the vesicle size, is reflected in the decay of the auto-correlation curve, whereas the amplitude is inversely proportional to the concentration of vesicles. In Figure 2f, the auto-correlation curves of a representative experiment are shown, while Figures 2g and h depicts the changes in vesicle diffusion time and particle numbers averaged from three independent experiments.

LUVs incubated with cBid or cBid/Bax showed a significantly shorter diffusion time and an increased concentration compared with the control sample (Figures 2g and h), which indicates a reduction in vesicle size as in the GUV experiments. Moreover, cBid and cBid/Bax increased the number of vesicles, suggesting vesicle fission. Bcl-xL inhibited both the changes in vesicle size and number (Figures 2g and h).

### cBid and Bax promote vesicle budding and fission

These data showed that cBid, Bax and Bcl-xL changed membrane structure beyond pore formation. The FCS experiments suggested vesicle fission involved. If this was the case, buds should be formed on the GUV surface, which we visualized with confocal microscopy. In absence of Bcl-2 proteins, the z-projections of GUVs showed a homogenous vesicle surface, but incubation with cBid and Bax induced bright dots on the surface (Figures 1c and e), which have been previously associated with membrane buds.^55, 56, 57^ In some cases we were able to image bud formation and fission (mainly happening towards the outside of the GUV's; Figure 3a). However, due to its rare and stochastic nature, bud formation and release was difficult to follow by imaging.

Compared with the control, images of GUVs incubated with cBid or cBid/Bax showed an increase in small floating particles (Figure 2a), likely related to released buds . In order to quantify the membrane material dissociated from the GUVs, the detection volume of the confocal microscope was placed above the GUVs and fluorescence intensity traces were recorded. Diffusing vesicles appear as peaks in the intensity traces (Figure 3c). To separate peaks corresponding to bud particles from noise and background fluorescence, we set a threshold 10 times bigger than the baseline of background fluorescence (Supplementary Information). In parallel, we followed the changes in GUV size, as budding should correlate with a decrease in GUV size (Figure 3b).

Compared with the control, cBid or cBid/Bax provoked a clear and concentration-dependent increase in the number of peaks (Figure 3d), which correlate with lipid particles being extracted from the peaks showed a broad range of intensities (Figure 3e) and residence times (Figure 3c) indicating particles of different sizes. Notably, the presence of Bcl-xL had a clear inhibitory effect on the process (Figure 3d).

Bigger vesicles need longer times to cross the focal volume and are brighter due to the larger amount of fluorophores they contain. We calculated the average times differently sized vesicles need to cross the focal volume and the expected brightness of those particles (Supplementary Information). From the imaging (Figures 1d and e), we know that the buds have mainly a radius below 1 *μ*m, which would correspond to residence times <0.3 s and brightness <1000 kHz (Supplementary Information), which fits well with the experimental data (Figures 3c and e). A few brighter particles with longer residence times are detected, which are mainly present upon incubation with cBid/Bax (Figure 3e). These particles could be tubes, GUVs or tethered vesicles with slow diffusion.

### cBid and Bax bind to highly curved membranes

**Figure 4:**
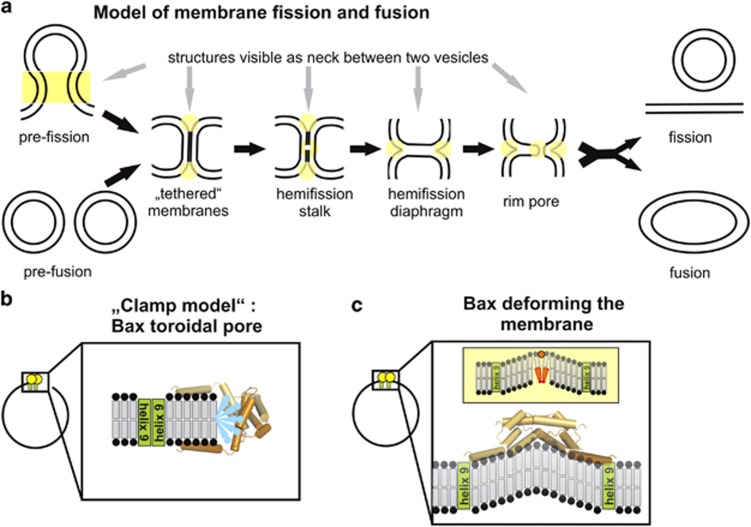
Model of membrane fission and fusion, and possible scenario on how Bax reorganizes the membrane. (**a**) Model of membrane fission and fusion was adapted from Frolov and Zimmerberg.^35^ Fission and fusion are lipid-mediated events that are mainly described theoretically^35, 36, 37, 38^ and are proposed to happen via the same intermediates. The membrane that fuse or divide needs to come into close contact, allowing the formation of a so-called ‘hemi-fission/fusion stalk'. Stalk formation is proposed to start via exchange of lipid tails at the contact area of the two outer membrane leaflets. This creates a connection that may grow into a ‘hemi-fission/fusion diaphragm'. However, this process is energetically unfavored and needs the exclusion of water molecules from the contact area. Therefore, the membranes need to come in close contact, which are visible as membrane tethering. To resolve the ‘hemi-fission/fusion diaphragm' into the fused or divided membranes, the opening of a transient pore (called the ‘toroidal rim pore') has been proposed. As a result, membrane fusion and fission proceed via several unstable structures containing highly curved membranes (highlighted in yellow), which can happen spontaneously, but in cells is usually stabilized by dedicated proteins. (**b**) ‘Clamp model' of membrane embedded Bax stabilizing a toroidal pore.^16^ Toroidal pore formation is eased when the pore rim is formed by lipid having an inverted cone shape (indicated in blue). (**c**) Suggestions how Bax may induce membrane curvature. Upper example: the shallow insertion of one hydrophobic helix (shown as orange circle) acting like a wedge in the membrane that induces curvature. Here, cone-shape lipids (shown as orange lipid) like cardiolipin can stabilize the membrane curvature. Lower example: similar to BAR domains Bax could force the membrane to bend due to scaffolding. Here Bax is drawn according to the ‘in plane' model^18^

**Figure 5:**
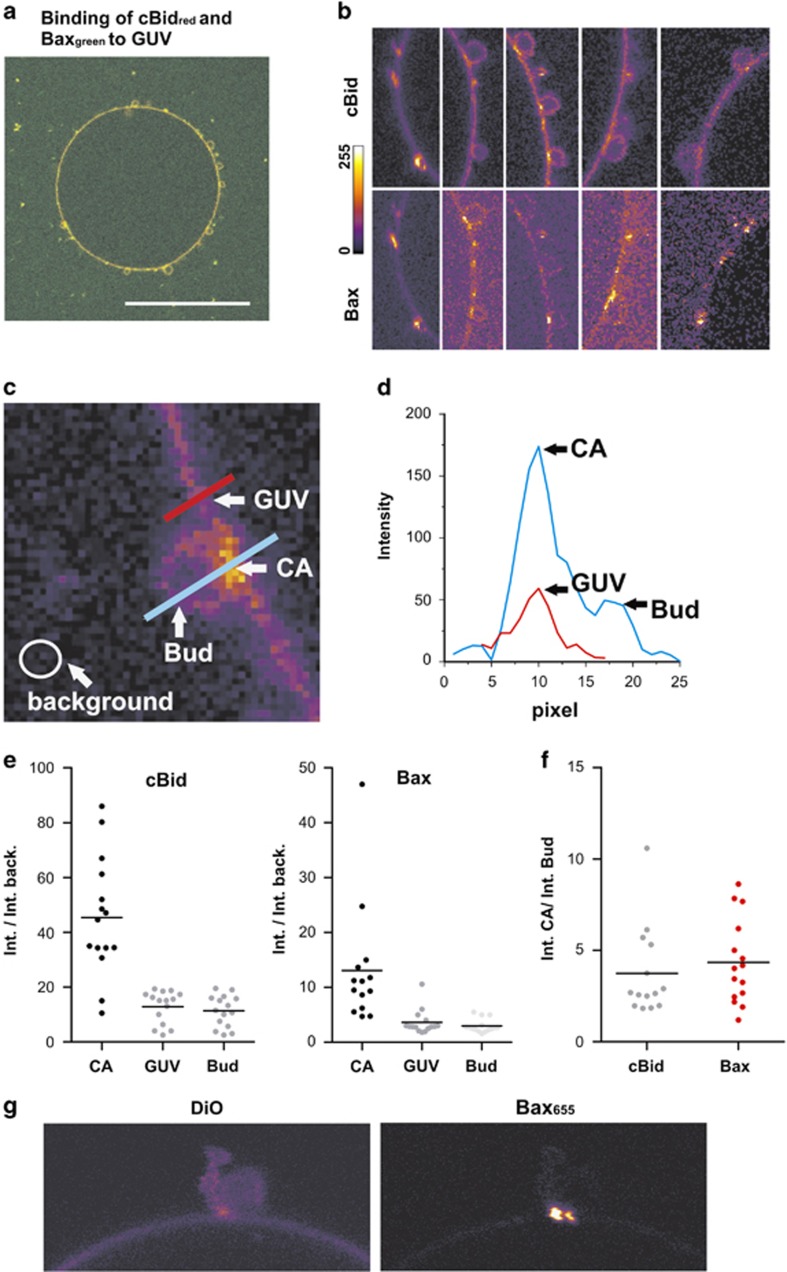
cBid and Bax bind preferentially to highly curved membranes. (**a**) Image of one GUV composed of 30% CL and 70% PC after 30 min incubation with 20 nM cBid_655_ and 40 nM Bax_488_. Shown is the color merge. Scale bar: 50 *μ*m. (**b**) Zoom-in's from (**a**) and other GUVs are shown to visualize that cBid and Bax bind preferentially at the highly curved interface between bud and GUV. A scale bar concerning the color code related to the pixel intensity is shown on the left side. (**c**) Example image on how the pixel intensities in the bud membrane, in the GUV membrane and in the contact area (indicated as CA) as well as the background are measured. The results are drawn in (**d**)–(**f**). (**d**) Display of the exemplary line scans shown in (**c**). (**e**) Pixel intensities for cBid and Bax at the contact area, the GUV and the bud (divided by the background intensity) from several GUV/bud pairs. (**f**) Ratio of the pixel intensity in the GUV/bud contact area compared with the pixel intensities at the GUV membranes. (**g**) Zoom-in from an exemplary GUV bud pair labeled with DiO and incubated with cBid unlabeled and 40 nM Bax_655_

Bud and GUV are connected by a highly curved neck region (Figure 4a) and toroidal pores are also characterized by highly curved membranes (Figure 4b). To compare the localization of cBid and Bax in curved versus flat parts of the membrane, GUVs enriched in cardiolipin (30% CL and 70% PC) were incubated with fluorescently labeled cBid and Bax. The label did not interfere with protein action^24^ and the lipid mixture was to be suitable to study pore formation and membrane binding.^24, 43^ The resolution of the confocal microscope was too low to discriminate whether the protein binds to the bud or the contact area of small buds. Therefore, the analysis was limited to a subpopulation of relatively big buds. cBid and Bax were found predominantly in the neck between vesicle and bud (Figures 5a–c). The fluorescence intensity was higher than expected for two membranes (Figures 5c and f), demonstrating that cBid and Bax were enriched in the neck region. To ensure that the bright areas contained membrane and were not protein aggregates, GUVs labeled with a green fluorescent dye and Bax_Atto655_ (activated by unlabeled cBid). This showed that the membrane is present at the neck and that Bax, but not the membrane dye was highly enriched in that area (Figure 5g).

### cBid/Bax induce membrane tethering and pore formation in LUVs visualized by cryo-EM

**Figure 6:**
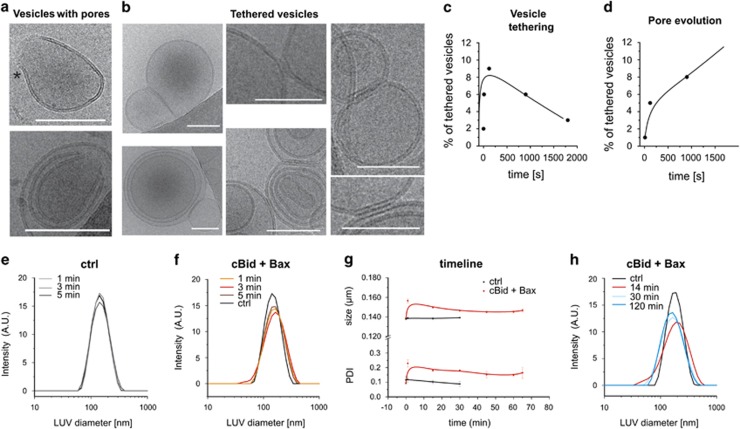
Morphological changes on LUVs upon incubation with cBid/Bax/Bcl-xL. (**a** and **b**) Cryo EM images of pore containing (asterisk indicates pore edge region with negative curvature as introduced by Gillies *et al.*^60^); (**a**) or tethered (**b**) vesicles. Scale bar: 75 nm. (**c** and **d**) Percentage of tethered (**c**) or pore containing (**d**) vesicles at the different time points gained from Cryo EM experiments. Final concentrations used for cyro EM: 1 *μ*M cBid; 2 *μ*M Bax; 2 mM lipids. For further information on the number of vesicles or experiments see Table 1. (**e**, **f** and **h**) Size distribution of control vesicles (**e**) or vesicles incubated with cBid and Bax (**f** and **h**) during different time points of DLS experiments. (**g**) DLS time course experiment following vesicle size and homogeneity over time. Here one representative experiment of three is shown. Final concentrations used for DLS: 20 nM cBid; 40 nM Bax; 40 *μ*M lipids

The spatial resolution of confocal microscopy does not allow following cBid/Bax-induced morphological changes at the membrane with molecular detail. To overcome this limitation, we used cryo electron microscopy (EM). As the ice layer of a cryo grid is typically a few-hundred nanometers thick, the micrometer-sized GUVs would be destroyed or strongly distorted by the blotting and we could not get images of them. Instead we used LUVs of 100–400 nm diameter (Figure 6e) with the disadvantage that the LUV's have a different curvature as the GUVs, the proteins might not act the same way.

Membrane pores induced by Bax with diameters between 10 and >100 nm were previously imaged using cryo EM.^15, 58, 59, 60^ We detected similar pores with negative lipid curvature at the pore edges, in line with a recent publication^60^ (Figure 6a). Like the previous studies,^15, 58, 59, 60^ we could not detect electron densities related to the proteins. This is compatible with our ‘clamp' model of membrane-embedded Bax, in which Bax lies on the membrane surface to stabilize the toroidal pore^16^ (Figure 4b) and could make it hard to discriminate electron density related to the protein from the membrane.

Aside from the pores, we observed a higher percentage of tethered vesicles in presence of cBid and Bax compared with controls (Figure 6b). Vesicle tethering is a necessary structural intermediate of membrane fission and fusion (Figure 4a), and was detected in EM studies of vesicle fusion.^61, 62^ As Drp1 has also been shown to induce membrane tethering,^40, 51^ it is reasonable to hypothesize that the membrane tethering induced by cBid/Bax might be related to membrane remodeling.

To study the temporal evolution of pore formation and tethering, we compared vesicles incubated 10 s to 60 min with cBid/Bax with control vesicles and counted the fraction of vesicles showing pores or tethering. To have a suitable number of vesicles the results from all experimental repetitions were merged (Table 1). Due to an insufficient number of LUVs, the 60-min sample was excluded from analysis. The amount of tethered vesicles increased during the first 5–10 min and decreased afterwards. In contrast, the fraction of pore containing vesicles increased over time. Thus, cBid and Bax seemed to promote transient membrane tethering before pore formation (Figures 6c and d; Table 1).

To back up the poor statistics of the cryo EM experiments, we performed bulk experiments using dynamic light scattering (DLS). In time course experiments, control vesicles without protein were constant in size, whereas cBid/Bax induced a transient increase in LUV size during the first minutes of incubation in line with vesicle tethering (Figures 6e and g; Supplementary Figure 1). At later time points, a new population of smaller vesicles was detected in agreement to vesicle fission (Figure 6h). As the increase in vesicle size was transient, vesicle tethering was likely not associated with membrane fusion.

In contrast to the cryo EM results, DLS and FCS showed no indication of vesicle loss upon 1–2 h incubation with cBid/Bax (Figures 2h and 6h). As cryo EM samples need to be transferred onto a grid and frozen before image acquisition, it could be that a specific loss of liposomes with membrane bound cBid/Bax happened due to changes in the surface properties of the vesicles. In this scenario, the number of pore containing and tethered vesicles would be underestimated, without affecting the conclusion of the experiment.

## Discussion

In cells, Bcl-2 proteins have roles in MOM permeabilization (by forming toroidal pores) and in alterations in mitochondrial dynamics.^7, 9, 63^ Although the molecular mechanisms involved are poorly understood, a connecting theme between both is the presence of locally highly curved and non-lamellar membrane structures. Here we have used minimal systems to disect the membrane activity of representative Bcl-2 proteins with a focus on membrane remodeling. The usefulness of such strategy is supported by the fact that most of our current understanding about membrane permeabilization, fission and fusion is derived from studies using membrane model systems,^21, 27, 61, 64, 65, 66, 67^ as the complexity of cells and the associated technical limitations currently prevent the study of these processes in depth *in vivo*.

In addition to permeabilization, cBid and Bax promoted the formation of tethered vesicles, buds on the GUV surface and release of lipid material from the membrane. Remarkably, both membrane permeabilization and budding activity could be specifically inhibited by Bcl-xL, indicative of the specific action of cBid and Bax. cBid and Bax were preferentially located at the neck region between GUV and bud, and showed the intrinsic ability to induce or stabilize membrane areas with high curvature and non-lamellar organization. As a consequence, these proteins reduced the energy required for membrane remodeling, which could have a role in the processes of pore formation and membrane fission and fusion.

What do the mechanisms of membrane budding, fission, fusion and pore formation have in common? Budding is the generation of a curved membrane region that can evolve to membrane fission. To start the fission process, the two membranes need to be very close together, which is usually achieved via membrane constriction, followed by the formation of a highly curved, non-lamellar neck region, which evolves to finally separate two membrane-enclosed compartments. Membrane fusion proceeds via the same structural intermediates but in opposite direction, and it is initiated via membrane tethering instead of constriction (Figure 4a). Main drivers of membrane scission are the members of the dynamin superfamily, whereas proteins such as the SNARE proteins are responsible for vesicle fusion.^68, 69^ Fusion and scission are favored by high local concentrations of shallowly inserted amphipathic protein helices that create local membrane curvature.^66, 68^ These amphipathic helices may be part of the fusion/fission protein structure, like in the case of the PH domain of dynamin, or of additional protein factors that participate in the process (like synaptotagmin in membrane fusion or epsin in fission).^62, 66, 70, 71^ Drp1 does not contain such a helix and likely needs additional cofactors to mediate mitochondrial fission.^49^ Interestingly, the stabilization of local membrane curvature is also crucial for toroidal pores.

Bax and cBid both contain amphipathic helices that have been proposed to superficially interact with membranes^16–18, 72^ and could induce local membrane curvature by shallow insertion. The bigger the area of amphipathic helix (helices) is with respect to rest of the protein, the stronger its effect on membrane curvature, which is additionally enhanced by protein oligomerization enhances.^66, 68, 73^ As cBid and Bax are small and have several amphipatic helices in membrane contact,^16, 17, 18, 72^ both have theoretically a strong curvature-inducting effect, which should be enhanced in Bax by oligomerization. In agreement with this, a peptide derived from Bax helix 5 reduced the energy cost associated with curved membranes.^44^

A second, not mutually exclusive possibility is that Bax stabilizes local membrane curvature by scaffolding. A large part of the structure of membrane-embedded Bax was recently solved.^16, 17^ Helices 2–5 of Bax form a rigid, slightly curved homo-dimer, with many hydrophobic and aromatic amino acids clustered at the interface proposed to be in membrane contact.^16, 17, 18^ Assuming this idea is correct, Bax could force its own curved shape onto the membrane as shown for BAR-domain proteins^74^ (Figure 4c).

Finally, we cannot discard that cBid and Bax stabilize local membrane curvature by recruiting lipids with intrinsic curvature that stabilize non-lamellar membrane structures.^35, 36, 68, 75^ Cardiolipin is a cone-shaped lipid that is important for membrane binding of cBid and Bax.^24, 76, 77^ The preferential interaction of cBid and Bax with this lipid could increase its local concentration and reduce the energy required for membrane remodeling.

In addition, our results demonstrate that Bcl-xL inhibits not only cBid/Bax induced pore formation but also vesicle budding and fission. Bcl-xL modulates Bax via different mechanisms: (i) Bcl-xL inhibits Bax activation by sequestering tBid;^24, 26^ (ii) Bcl-xL reduces the oligomerization state of Bax within membranes;^78^ and (iii) Bcl-xL decreases the membrane-bound population of Bax.^11, 12, 13^ In addition, our data support a novel mechanism by which Bcl-xL inhibits the activity of cBid and Bax by restraining their effects on membrane curvature. cBid alone is able to affect membrane structure and this activity is lost when Bcl-xL is present. As tBid adopts different conformations in the membrane^22, 79^ and forms tight complexes with Bcl-xL,^26^ it is tempting to speculate that, in complex with Bcl-xL, cBid and maybe even Bax capture conformations unable to induce membrane curvature. In any case, our findings reveal that Bcl-xL inhibits apoptosis by more complex mechanisms than thought so far.

The membrane remodeling activity described here for cBid and Bax may have physiological implications in the context of the cell. It was shown that tBid translocates to mitochondrial contact sites and reorganizes the mitochondrial structure at the cristae junctions and the contacts between inner and outer mitochondrial membranes.^80^ The ability of cBid to reorganize membrane shape reported here correlates very well with this.

Several studies report that Bax co-localizes at the MOM with Drp1 and Mitofusins that are involved in mitochondrial dynamics. Moreover, Bax is proposed to be involved in the regulation of mitochondrial dynamics.^28, 29, 30, 40^ The generation of local high curvature and the stabilization of non-lamellar membrane intermediates by Bax at the MOM fission and fusion sites could reduce the energy required for Drp1 and/or mitofusin-mediated membrane fission and fusion, thus promoting these processes. Given that the intrinsic pathway of apoptosis is accompanied by massive mitochondrial fragmentation mediated by Drp1, it is tempting to speculate that Bax could act as a cofactor of Drp1 to promote mitochondrial fission.

In summary, our work reveals that cBid and Bax are not only involved in membrane permeabilization but can also induce membrane remodeling. We propose that this is achieved mechanically by inducing or stabilizing membrane curvature, which supports a common molecular basis for their role in MOM permeabilization and in mitochondrial dynamics. In line with its function as cBid/Bax antagonist, Bcl-xL can inhibit membrane remodeling. Future research is needed to understand the complex molecular interplay between Bcl-2 proteins and the machinery regulating mitochondrial dynamics.

## Materials and Methods

### Protein production and labeling

Full-length mouse Bid, full-length human Bax and full-length human Bcl-xL were expressed in *E. coli* and purified as described in Bleicken *et al.*^15, 24^ and Suzuki *et al.*^81^ From Bid, cBid was cleaved and purified as described in Bleicken *et al.*^14^ Bovine Cyt c and Allophycocyanin (APC) were purchased at Sigma-Aldrich (Munich, Germany). Protein labeling was performed as described in Bleicken *et al.*^24, 43^ Human Drp1 was produced as a fusion protein with the calmodulin-binding protein as described in Ugarte-Uribe *et al.*^51^ GTPase activity and membrane binding of Drp1 were shown in Ugarte-Uribe *et al.*^51^

### Composition of the lipid mixtures

The lipid mixture mimicking the MOM composition was prepared as in^14, 16, 21^ with 49% egg l-α-phosphatidyl-choline, 27% egg l-α phosphatidyl–ethanolamine, 10% bovine liver l-α-phosphatidyl–inositol, 10% 18 : 1 phosphatidyl–serine and 4% cardiolipin (all percentages mol/mol). In addition, a lipid mixture composed of 30% cardiolipin and 70% phosphatidyl–choline (mol/mol) was used.^24, 43^ All lipids were purchased from Avanti polar lipids (Alabaster, AL, USA). The lipidic dyes DiD (1,1′-dioctadecyl-3,3,3′,3′-tetramethylindodicarbocyanine, 4-chlorobenzenesulfonate), DiI (1,1′-dioctadecyl-3,3,3′,3′-tetramethylindocarbocyanine perchlorate) and DiO (3,3′-dilinoleyloxacarbocyanine perchlorate) (all Thermo Fisher Scientific, Waltham, MA, USA) were used to visualize membranes in the confocal microscope.

### LUV preparation

The dried lipid mixtures was dissolved in buffer (150 mM NaCl, 20 mM tris pH 7.5) and prepared as described in Bleicken *et al.*^14, 15^ Briefly, mixing of the lipid film and buffer was accompanied by five cycles of freezing and thawing. Afterwards the lipid solution was passed 31 times through a membrane (200 or 400 nm pore size) using an extruder from Avestin (Mannheim, Germany).

### GUV permeabilization and GUV size determination experiments

GUVs were produced by electroformation and the experiments were done as described in Bleicken *et al.*^24, 82^ Briefly, 10 *μ*g lipid mixture dissolved in chloroform were spread on platinum electrodes in the electroformation chamber and allowed to dry, before immersion in 300 mM sucrose. Electroformation proceeded for 2 h at 10 Hz, followed by 30 min at 2 Hz. Cyt c_488_, APC and the proteins of interest were mixed in LabTec chambers (Thermo Fisher Scientific) with buffer (150 mM NaCl, 20 mM tris, pH 7.5) at the desired concentrations. Afterwards 75 *μ*l of the GUVs suspension was added to get a final volume of 300 *μ*l. Imaging was performed after 45–90 min incubation at room temperature, as indicated in the figure. The samples were imaged using a LSM710 confocal microscope with a C-Apochromat × 40 NA. 1.2 water immersion objective (Zeiss, Jena, Germany) with laserlines to excitate at 488, 561 or 633 nm. A spectral beam guide was used to separate emitted fluorescence. Images were processed with ImageJ (http://rsbweb.nih.gov/ij/) or a homemade analysis software^48^ detecting the filling and the size of each GUVs. Per sample well 200–1500 GUVs were analyzed.

The degree of GUV filling was calculated as:


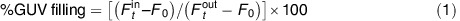


where *F*_*t*_^in^ and *F*_*t*_^out^ are the average fluorescence intensities inside and outside a GUV at time *t*, and *F*_0_ is the background fluorescence. We arbitrarily set the threshold for classifying GUVs as non-permeabilized for <20%. The experiments were set up as a way that the lipid to Bax ratio was ~500 : 1 or bigger. To make sure that the samples were statistically different, *P*-values were calculated by performing *t*-tests using the Graph Pad prism software.

### Fluorescence correlation spectroscopy experiments on LUVs and fluorescence burst analysis on GUVs

#### Experiments on LUVs

LUVs mimicking the MOM composition (and <0.05% DiD) were incubated with Bcl-2 proteins for 120 min at RT before solution FCS experiments were performed using a LSM710 confocal microscope equipped with a Confocor3 for FCS measurements, a C-Apochromat × 40 NA. 1.2 water immersion objective and laser to excite at 633 nm (Zeiss). The protein concentrations were chosen so that the lipid to Bax ratio was at least 500 : 1. Each sample was measured at least 300 s to assure sufficient data points to generate auto-correlation curves. To calculate the *t*_D_, the data were fitted using equations 2 and 3 assuming three-dimensional diffusion.


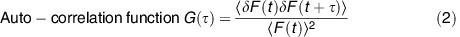



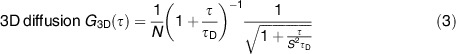


*Experiments on GUVs* For the burst analysis, the LSM710 with Confocor3 was used as mentioned earlier. GUVs mimicking the composition of the MOM were incubated at RT with Bcl-2 proteins in the absence of size marker proteins (to avoid noise due to channel cross talk). After incubation, images were taken to detect the mean GUV size. Moreover, fluorescence intensity traces were collected at three different position of the sample (each for 200 s and 60–80 *μ*m above the glass; laser power: 0.5% pinhole: 2 Airy units; [DiI]: 0.02%). Thereby, 600 000 data points per sample and experiment were collected. From the traces, the number of peaks with fluorescence intensity as bigger as 10-fold than the background intensity (mean count rate background: ~3 kHz) were counted and compared for different samples. More information is given in the Supplementary Information.

### Dynamic light scattering experiments

DLS experiments were performed on a zetasizer instrument (Malvern, Malvern, UK). Samples contain vesicles (20 *μ*M lipids) with or without 20 nM cBid and 40 nM Bax (attenuation 9 or 10, 10–16 runs per measurement). Experiments were done at RT.

### Cryo EM experiments

For sample preparation vesicles (1 mM lipids) were incubated at 37 °C with 1 *μ*M cBid and 2 *μ*M Bax for 10 s to 60 min and afterwards 5 *μ*l of the sample was transferred on before glow discharged (3 s) 2/2 Quantifoil grids. Grids with sample droplets were blotted and frozen using FEI Vitrobot (Hillsboro, OR, USA) at 4 °C and 100% humidity for 8–10 s. The grids were observed in a Titan Krios microscope (FEI) equipped with a Quantum 963 SE energy filter operated at 200 kV and LN temperature. Zero-loss filtered pictures were taken at × 64 000 magnification using a 2 k Ultrascan Quantum CCD camera.

Images were taken using the leginon software in a semi-automated procedure. After obtaining a grid map ( × 135) selected squares were imaged ( × 480) and a group of holes were selected for imaging at × 4300. At that magnification liposomes were visible and 1–4 positions were chosen for final acquisition. Pictures were taken with a dose of ~10 e-/Å^2^ at a defocus of ~1 *μ*m.

## Figures and Tables

**Table 1:** Cryo EM image analysis

	**Number of vesicles**	**% Unilamilar vesicles**	**% Tethered vesicles**	**% Pores**
ctrl	924	70	2	1
cBid/Bax 10 s	988	69	6	1
cBid/Bax 2 min	722	69	9	5
cBid/Bax 15 min	745	70	6	8
cBid/Bax 30 min	327	86	3	12

## Abbreviations

APC: Allophycocyanin
FCS: fluorescence correlation spectroscopy
Cyt c: Cytochrome c
DLS: dynamic light scattering
EM: electron microscopy
GUVs: giant unilamellar vesicles
LUVs: large unilamellar vesicles
MOM: mitochondrial outer membrane
