# Supplementary material for: cBid, Bax and Bcl-xL exhibit opposite membrane remodeling activities
Source: Cell Death Dis. 2016 Feb 25;7(2):e2121–. doi: 10.1038/cddis.2016.34 (PMC4849160; doi:10.1038/cddis.2016.34)
Supplement: Supplementary Information [file cddis201634x1.docx]

**Supplemental Information**

**Opposite functions of cBid, Bax and Bcl-xL in membrane remodeling processes associated with membrane fission and pore formation**

Stephanie Bleicken^1,2,3^, Götz Hofhaus^4^, Begoña Ugarte-Uribe^1,2,3^, Rasmus Schröder^4^, Ana J. García-Sáez^1,2,3^*


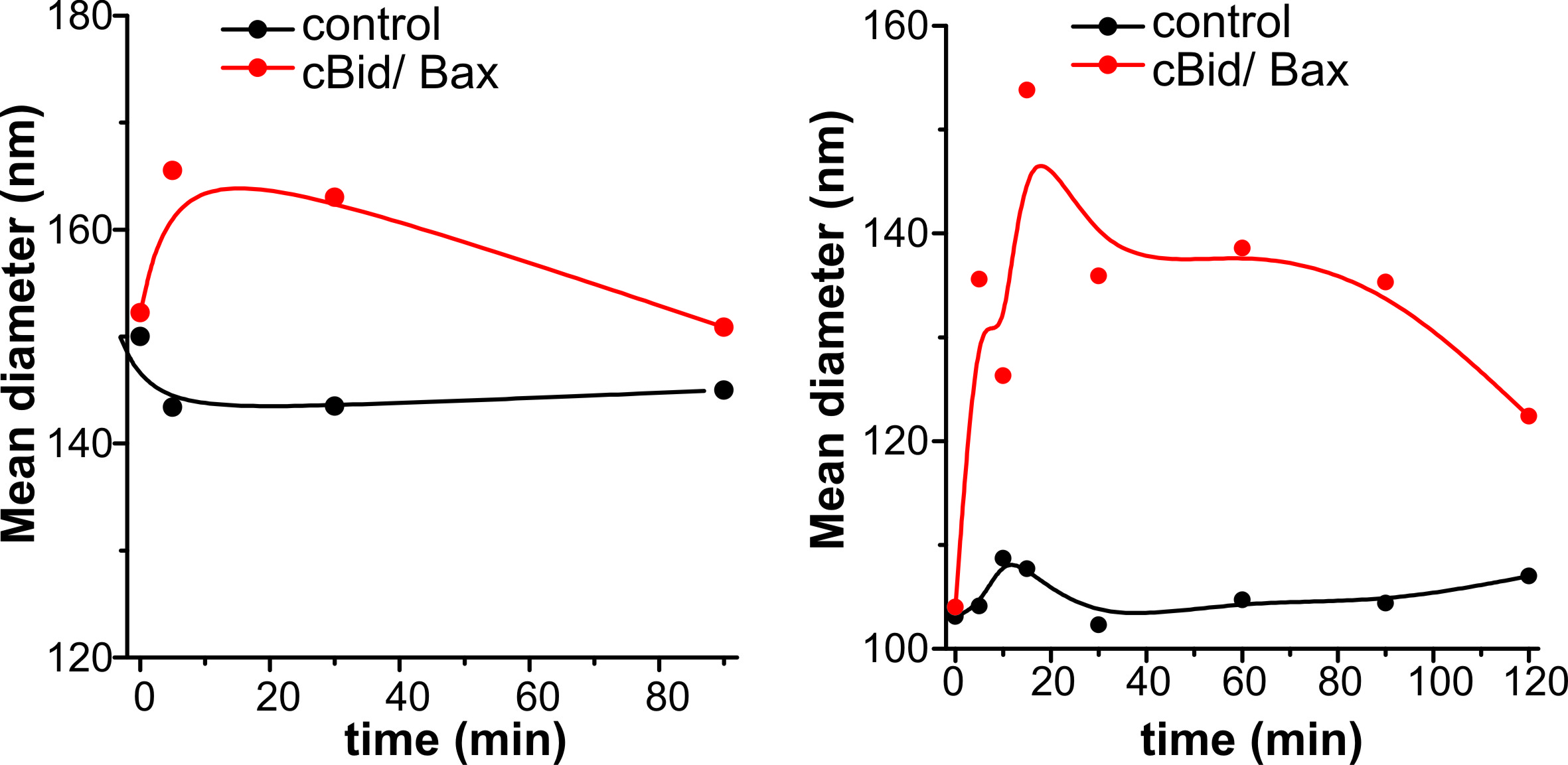


**SUPPLEMENTAL FIGURE 1: Repetitions of dynamic light scattering experiments**

Repetitions DLS time course experiment on vesicle size shown in Figure 6G.

**Threshold determination of the fluorescence Intensity traces shown in Figure 3:**

We first detected the baseline of fluorescence count rate for DiI, which is $<$3kHz. To determine the threshold, the brightness of single DiI fluorophores was measured. Under our experimental conditions, the count rate of single DiI molecules is below 5 kHz. Thus the baseline detected in the experiments presented in Figure 3 in due to single fluorophores and noise. As DiI is a membrane dye, it is hydrophobic and aggregates in absence of liposomes in buffer. Therefore, DiI cannot be studied in buffer and we performed measurements in ethanol instead and used them to estimate a suitable threshold to separate the background from peaks due to vesicle passage through the focal volume. We diluted the DiI in ethanol until the intensity trace has a similar count rate as the background of the real experiment. From those data we calculated the mean, standard deviation and SEM, which should be similar to the experimental baseline. We got a mean of 2.75 kHz, a standard deviation of 1.89, and the SEM was 0.01. Based on these values, we set the threshold to 30, which is > 10 fold the baseline and it is the mean plus >14 fold the standard deviation. This should remove all noise and influence of non-membrane bound fluorophores, so that we only detect vesicle signal.

**Theoretically expected brightness and residence times of different sized vesicles in intensity traces**

In the fluorescence intensity traces, all fluorescent particles that move through the focal detection volume of the confocal microscope are detected. The detection volume has a size of about one femtoliter. In his particular experiments we used a pinhole of two airy units and with calibration measurements we calculated the diameter of the focal volume in x and y direction to be ~0.9 µm and the structural parameter to be ~7. We measured a total of 600s per experiment, which come from three 200s traces, taken in different areas of the sample. The focal volume was placed clearly above the GUVs settled on the bottom of the observation chamber so that at only diffusing particles were detected.

The GUVs analyzed with our software are mainly between 4-100 µm in diameter and therefore bigger as the focal volume. The detected buds on the GUV surface were mainly smaller than 1 µm diameter and therefore in the size range of the focal volume or smaller. All particles in the size range of the focal volume or smaller should give rise to one peak when passing the focal volume. In contrast a 4-100 µm GUV would pass the focal volume most likely twice. The bigger the vesicle is, the longer its residence time in the focal volume. Moreover, bigger vesicles contain more fluorophores and therefore should have a higher brightness. Based on the calibration measurements, we could calculate the average residence time that different sized vesicles should need to cross the focal volume. Examples for this calculation are given in Supplemental Table 1. In line with the experimental results, all particles with a residence time below 0.3 s should be buds and indeed almost all peaks had such a short residence time.

Additionally, we detected some very slow defusing objects (tD>1s) that are rare and almost only present when cBid and Bax are incubated with the vesicles. They could correspond to tubes, tethered vesicles or big multilamellar vesicles.

**Supplemental Table 1: Calculation on the residence times of differently sized vesicles in the focal volume.**

| **radius of a vesicle** | **Calculated Diffusion coefficient D in µm^2^/s (calculated using eq.1)** | **Calculated residence time t_D_ in seconds (calculated using eq.2 ^1,2^)** |
| --- | --- | --- |
| 50 | 4.35 | 0.012 |
| 100 | 2.17 | 0.023 |
| 500 | 0.44 | 0.115 |
| 1000 | 0.22 | 0.23 |
| 4350* | 0.05 | 1 |

**Equation 1: D=µk_B_T ;Equation 2: D=ω_0_^2^/4t_D_; with µ being the particle velocity; T being the temperature; k_B_ being the boltzman constant; ω_0_ being the radius of the focal volume in the *x* and *y* directions; *D* being the diffusion coefficient; *t_D_* being the average time the fluorophore stays in the focal volume we also call this residence time in the focal volume. * This GUV should pass the focal volume in a way giving rise to two peaks.**

As we know the DiI concentration added to the lipid mixture (~0.02% in this experiment), the count rate of one DiI molecule (~4kHz) and the area of one lipid in the bilayer, we can calculate the brightness that would be expected for different sized vesicles. As the focal volume has a diameter of 1 µm, we consider only vesicles up to this size, as bigger particles will be only partly within the focal volume. We now show in Supplemental Table 2 different particle types and their calculated brightness.Based on this consideration, the majority of vesicles would be smaller than 0.5 µm. This is in line with the calculations of the residence times and with the images of buds detected on the surfaces of GUV (mainly <1 µm diameter) incubated with cBid and Bax (as shown in Figure 1DE). As a result, the traces detected experimentally fit well with the size expectations for released buds.

**Supplemental Table 1: Calculation on the brightness expected for different sized vesicles.**

| **Diameter of an unilamilar vesicle** | **Vesicle surface**  **(using eq.3)** | **Number of lipids per vesicle (considering ^a^)** | **Dye molecules per vesicle (considering ^b^)** | **Expected brightness of the vesicle (considering ^c^)** |
| --- | --- | --- | --- | --- |
| **100 nm** | **31400** | **88450** | **17** | **68** |
| **250 nm** | **78500** | **221125** | **44** | **176** |
| **1000 nm** | **314000** | **884500** | **170** | **680** |

**Equation 3: Vesicle surface = d(_vesicle_)² * π; ^a^: area of one lipid : 0.71 nm²; ^b^: Dil concentration: 0.02%.**

**^c^: Brightness of one DiI molecule under the given experimental condition ~4kHz.**

**References**

1 Ries, J., Weidemann, T. & Schwille, P. in *Comprehensive Biophysics* Page 210-245 (Elsevier, 2012).

2 Cosentino, K., Bleicken, S. & García-Sáez, A. J. Analysis of membrane-protein complexes by single molecule methods *Bookchapter in press* (2015).
